# Supplementary material for: The Use of Reproductive Indicators for Conservation Purposes: The Case Study of Palinurus elephas in Two Fully Protected Areas and Their Surrounding Zones (Central-Western Mediterranean)
Source: Biology (Basel). 2022 Aug 7;11(8):1188. doi: 10.3390/biology11081188 (PMC9404957; doi:10.3390/biology11081188)
Supplement: Supplementary file 1 [file biology-11-01188-s001.zip › biology-1794157-supplementary.pdf]

The use of reproductive indicators for conservation purposes: the case study of *Palinurus elephas* in two fully protected areas and their surrounding zones (Central-Western Mediterranean).

Cristina Porcu[1], Laura Carugati[1], Andrea Bellodi[1], Pierluigi Carbonara[2], Alessandro Cau[1], Danila Cuccu[1], Faustina Barbara Cannea[3], Martina F. Marongiu F.[1], Antonello Mulas[1], Alessandra Padiglia[3], Paola Pesci[1], Maria C. Follesa[1].

[1]Dipartimento di Scienze della Vita e dell'Ambiente - Sezione Bio-ecologia Marina -Università di Cagliari, 09126 Cagliari, Italy

[2]COISPA Tecnologia & Ricerca, Stazione Sperimentale per lo Studio delle Risorse del Mare, via dei Trulli 18-20, Bari, (BA), 70126, Italy

[3]Dipartimento di Scienze della Vita e dell'Ambiente - Macrosezione Biomedica -Università di Cagliari, 09126 Cagliari, Italy

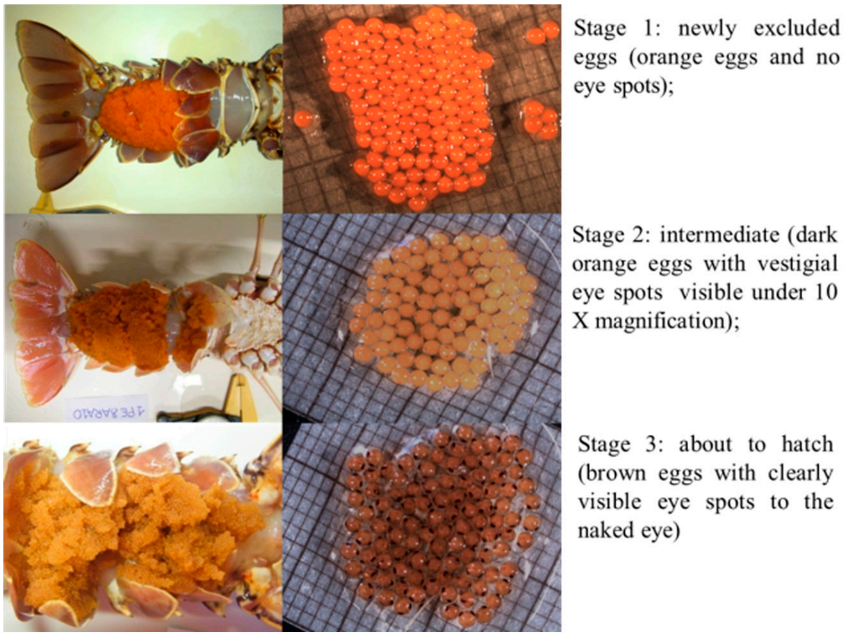

Figure S1. Different stage of egg development in *Palinurus elephas*.

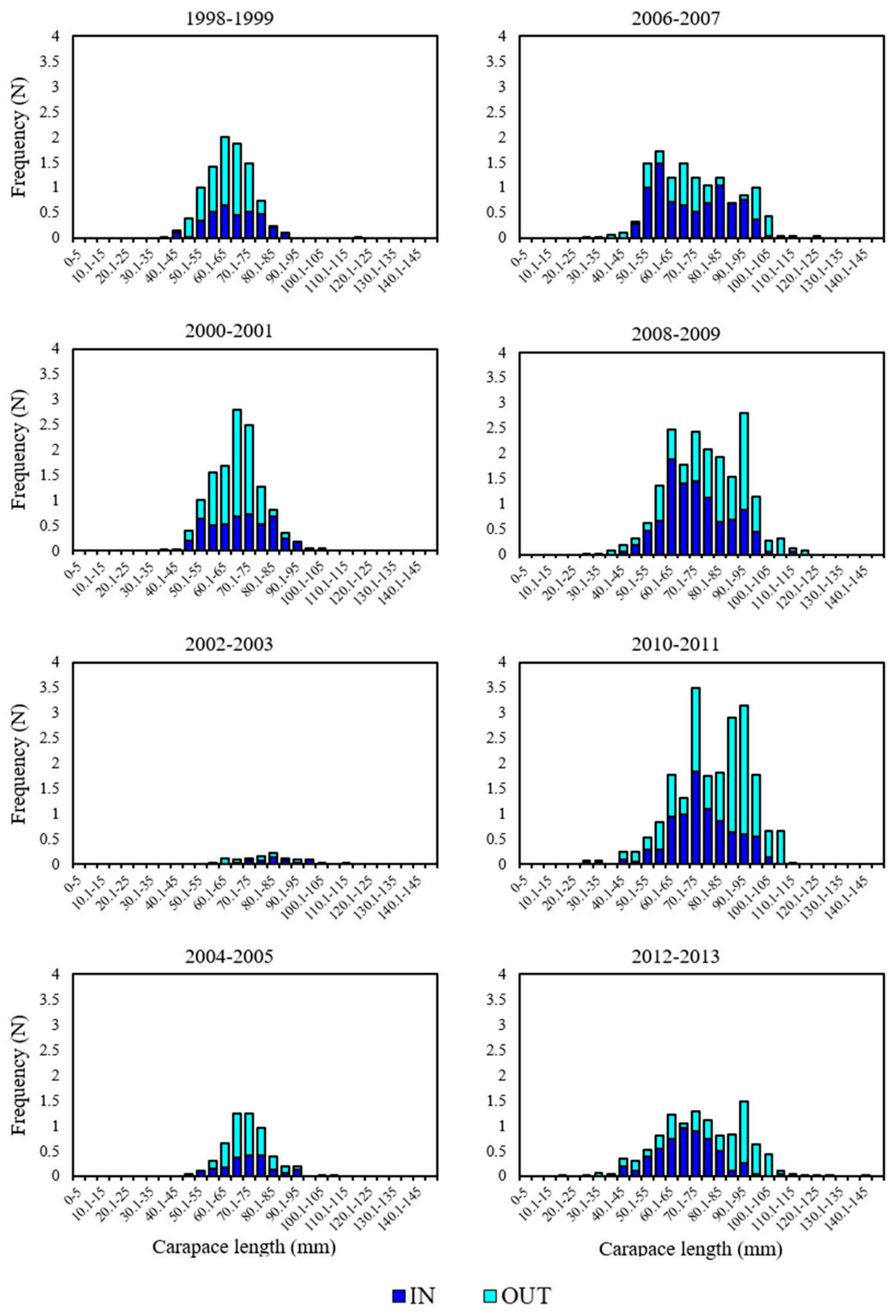

Figure S2. Size-frequency distribution (number) standardized to CPUE of female *P. elephas* inside (IN) and outside (OUT) Su Pallosu FPAs. Data combined in two years.

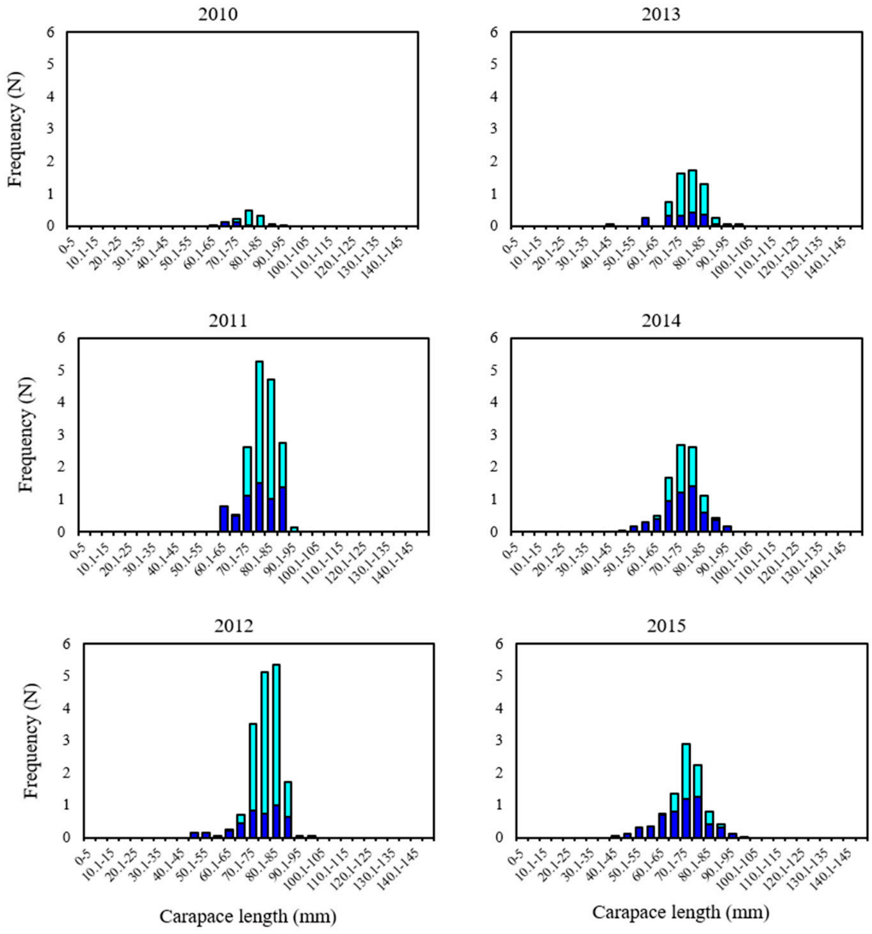

Figure S3. Size-frequency distribution (number) standardized to CPUE of female *P. elephas* inside (IN) and outside (OUT) Buggerru FPAs for each year analyzed.

Table S1. Output from the PERMANOVA analysis (main test) testing for differences in average CPUE and CL of mature females. Significant Monte Carlo procedure p-values [P(MC)] are reported in bold.

CPUE					CL				
**Source**	**df**	**MS**	**Pseudo-F**	**P(MC)**	**Source**	**df**	**MS**	**Pseudo-F**	**P(MC)**
**Su Pallosu**					**Su Pallosu**				
Time	7	46.08	6.5828	**0.001**	Time	7	1.61	47.52	**0.001**
FPA	1	27.878	27.878	**0.001**	FPA	1	0.199	5.88	**0.021**
Time X FPA	7	90.133	12.876	**0.001**	Time X FPA	7	12.45	12.45	**0.001**
**Buggerru**					**Buggerru**				
Time	5	1.844	3.871	**0.003**	Time	5	0.225	34.159	**0.001**
FPA	1	4.359	9.148	**0.003**	FPA	1	0.708	107.42	**0.001**
Time X FPA	5	1.615	3.390	**0.007**	Time X FPA	5	0.0381	5.782	**0.001**

Table S2. Output from the PERMANOVA analysis (main test) testing for differences in average CPUE and CL of mature females inside and outside Su Pallosu and Buggerru. Significant Monte Carlo procedure p-values [P(MC)] are reported in bold.

CPUE					CL				
**Source**	**df**	**MS**	**Pseudo-F**	**P(MC)**	**Source**	**df**	**MS**	**Pseudo-F**	**P(MC)**
**Su Pallosu IN**					**Su Pallosu IN**				
Time	7	1.503	5.0064	**0.001**	Time	7	0.212	6.114	**0.001**
**Su Pallosu OUT**					**Su Pallosu**				
Time	7	32.259	34.432	**0.001**	Time	7	4.523	134.67	**0.001**
**Buggerru IN**					**Buggerru IN**				
Time	5	1.573	3.7633	**0.013**	Time	5	190.35	1.9247	0.108
**Buggerru OUT**					**Buggerru IN**				
Time	5	2.6982	5.5211	**0.001**	Time	5	0.3253	94.427	**0.001**

Table S3. Output from the pairwise comparison testing for differences in Index of egg production among years inside (IN) and outside (OUT) portion of FPAs.

Egg production			
**Buggerru**		**Su Pallosu**	
Time	p-value	Time	p-value
2010	0.022*	1998	0.32
2011	0.215	1999	0.289
2012	0.045*	2000	0.615
2013	0.070*	2001	0.432
2014	0.001*	2002	0.20
2015	0.001*	2003	0.347
		2004	0.239
		2005	0.245
		2006	0.0061*
		2007	0.012*
		2008	0.001*
		2009	0.005*
		2010	0.001*
		2011	0.001*
		2012	0.003*
		2013	0.002*
